# iPSC-Derived Organoids as Therapeutic Models in Regenerative Medicine and Oncology

**DOI:** 10.3389/fmed.2021.728543

**Published:** 2021-10-13

**Authors:** Ali G. Turhan, Jinwook W. Hwang, Diana Chaker, Albert Tasteyre, Theodoros Latsis, Frank Griscelli, Christophe Desterke, Annelise Bennaceur-Griscelli

**Affiliations:** ^1^INSERM UA/09 UMR-S 935, Université Paris Saclay, Villejuif, France; ^2^ESTeam Paris Sud, Université Paris Saclay, Villejuif, France; ^3^APHP Paris Saclay, Department of Hematology, Hopital Bicetre and Paul Brousse, Villejuif, France; ^4^INGESTEM National iPSC Infrastructure, Villejuif, France; ^5^CITHERA, Centre for IPSC Therapies, INSERM UMS-45, Genopole, Evry, France; ^6^Université Paris Descartes, Faculté Sorbonne Paris Cité, Faculté des Sciences Pharmaceutiques et Biologiques, Paris, France

**Keywords:** induced pluripotent stem (IPS) cell, organoid, cancer, drug discovery, regenerative medicine

## Abstract

Progress made during the last decade in stem cell biology allows currently an unprecedented potential to translate these advances into the clinical applications and to shape the future of regenerative medicine. Organoid technology is amongst these major developments, derived from primary tissues or more recently, from induced pluripotent stem cells (iPSC). The use of iPSC technology offers the possibility of cancer modeling especially in hereditary cancers with germline oncogenic mutations. Similarly, it has the advantage to be amenable to genome editing with introduction of specific oncogenic alterations using CRISPR-mediated gene editing. In the field of regenerative medicine, iPSC-derived organoids hold promise for the generation of future advanced therapeutic medicinal products (ATMP) for organ repair. Finally, it appears that they can be of highly useful experimental tools to determine cell targets of SARS-Cov-2 infections allowing to test anti-Covid drugs. Thus, with the possibilities of genomic editing and the development of new protocols for differentiation toward functional tissues, it is expected that iPSC-derived organoid technology will represent also a therapeutic tool in all areas of medicine.

## Introduction

Organoids are tridimensional assembly of cells, mimicking organ-like features generated *in vitro* under specific cues (1). Their ability of self-organization *in vitro* under defined conditions allows their development for several days or weeks depending on the conditions of culture. There are now extensive data showing that these structures can recapitulate some of the features observed in adult organs, opening therefore major perspectives for their use in disease modeling, precision oncology and perhaps in the future as tools of regenerative medicine. This technology has also the potential to replace animal experiments as theoretically any tissue can be generated *in vitro*.

Currently, organoid-like structures have been successfully generated from several human tissues (1). These include essentially heart, digestive system, liver, brain, lung, and kidney organoids. These structures have been used to generate either healthy or diseased tissues, allowing to compare the behavior of different cell populations contributing to the organoid under *in vitro* conditions. They have provided important clues for the identification of new signaling pathways and novel targets especially in precision oncology. However, they require obviously a biopsy which might be difficult to obtain. The advantage of these organoids generated from cancer tissue biopsy is obviously the possibility of obtaining more precise information with regard to the tumor microenvironment as this is discussed below. On the other hand, primary cancer-derived organoids are not amenable to genome editing or extensive long-term cultures. One approach to circumvent these obstacles and which is currently in development is the use of iPSC technology to manufacture different organoids which is the subject of this review.

## iPSC-Derived Organoids

The pioneering work of S. Yamanaka which led to the revolutionary iPSC technology allows the reprogramming of an adult somatic cell toward an embryonic state similar to embryonic stem cells (ESC). Since their initial description in 2006 in mice (2) and in 2007 in human cells (3), iPSC are increasingly studied in stem cell research and more recently in the therapeutic arena by the possibility of generating differentiated cells for therapeutic purposes (4). More recently, the attention was focused on the possibility of generating from pluripotent stem cells (either iPSC or ESC) organ-like structures called “organoids” initially described from the adult tissue samples (1).

The organoid field has emerged from pioneering work of the group of Hans Clevers which has shown initially the possibility of generating gut organoids from Lgr5+ stem cells (5). These findings have now been reproduced and extended to other tissues and organoids have been obtained from several adult tissues. More recently, IPSC-derived organoids came into the forefront of stem cell research, due to the fact that as compared to adult tissue-derived organoids, they offer the possibility to combine the self-organization potential of iPSCs and the possibility of directing these cells toward potentially to any organ-specific differentiation (6).

Based on the events of human embryonic intestinal development, Spence at al first showed the possibility of generating intestinal organoids using a series of successive growth factor additions allowing endoderm induction, patterning and morphogenesis from human embryonic stem cells (H1, H9) and from four lines of iPSC. Interestingly, the 3D intestinal structures showed functional features of intestinal epithelium such as absorption and exocrine functions (7). Similarly, using self-organizing embryonic stem cells, Nakano et al. (8) have shown the possibility of generation of 3D optic cups.

*in vitro* models of brain development have also been possible with the advent of iPSC-derived organoids technology. In 3D culture systems, it was possible to generate mini-brains with highly specialized zones and structures such as cortex and radial glial cells and to model human microcephaly (9). The initial methodology has now progressed to the stage where it is possible to generate highly specialized cells such as oligodendrocytes and astrocytes (10) as well as long-term culture procedures of cerebral structures leading to highly specialized advanced brain organoids to study later stages of neural development (11). Finally the possibility of generating separately different parts of human brain has been described (12).

In the field of kidney development and kidney diseases, iPSC-derived kidney organoids allowed the possibility of generating highly specialized structures such as distal and proximal tubules as well as glomeruli with podocytes with a transcriptomic features similar to that of human fetal kidneys (13). iPSC-derived kidney organoid technology is of interest not only for gaining pathophysiologic insights but also to determine the effects of drug development in the transplant setting, for instance to evaluate the toxicity of tacrolimus (14).

**Figure 1:**
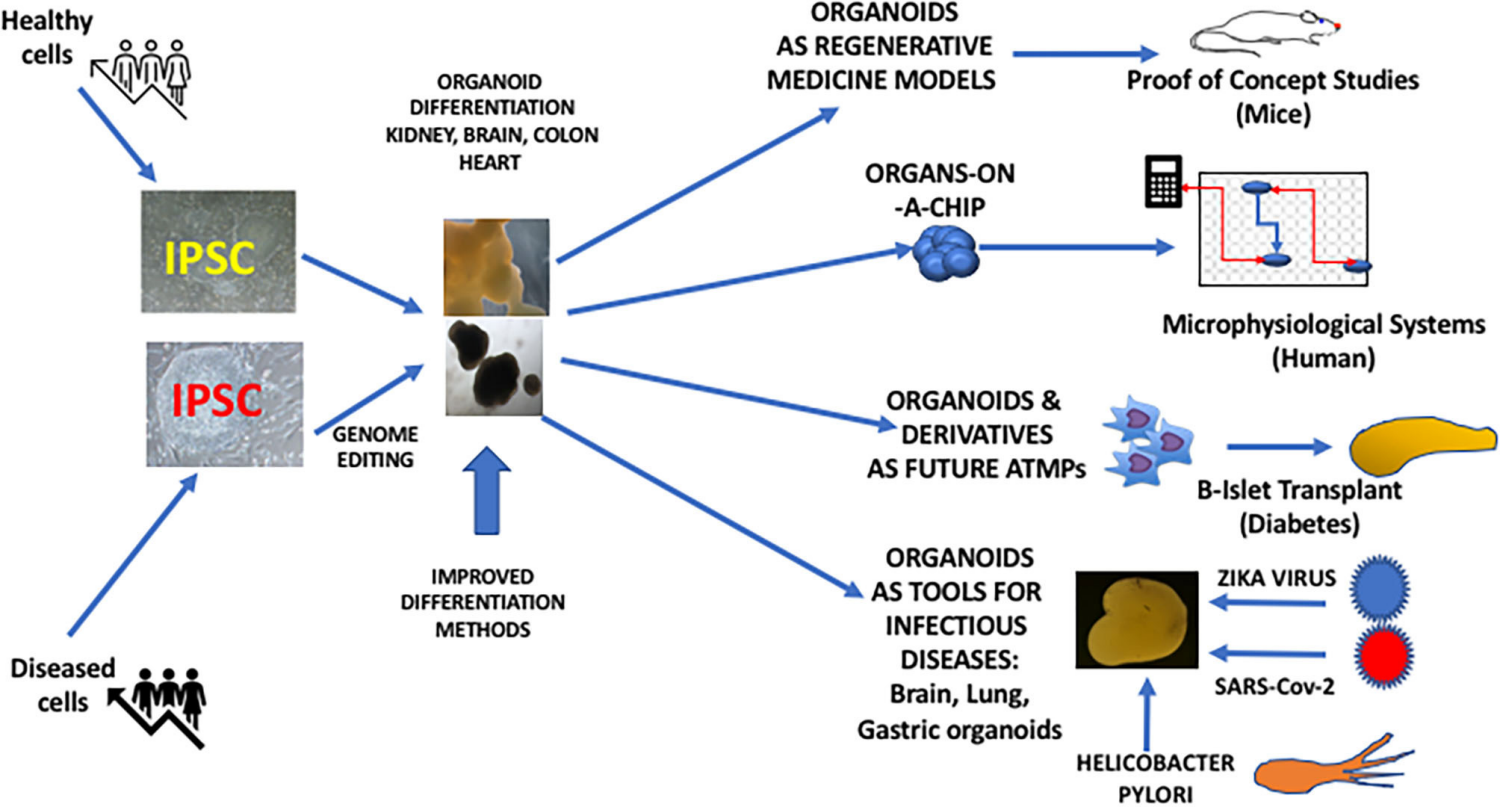
iPSC-derived organoids and their potential use in Regenerative Medicine.

The complex structure of the liver can also be reproduced using iPSC-derived organoid technology. One of these studies has shown the possibility of obtaining transplantable 3D hepatic buds with functional activities by the combined culture of iPSC-derived endoderm directed toward hepatic differentiation along with mesenchymal stem cells and the endothelial HUVEC cell line (15) Hepatic organoids can also be obtained from normal or patient-derived iPSC to model human hepatic diseases (16, 17). Although adult liver tissue can be targeted to generate hepatic organoids, iPSC-derived liver organoids can have a potential advantage of their expansion ability, which can be of interest for toxicology purposes allowing to screen large numbers of compounds in the industrial setting (17). ESC and iPSC-derived cardiac structures can be obtained with highly reproducible methods, giving rise to contractile structures including the possibility of morphological compartmentations such as cardiac chambers (18). This technology represents also an important tool for drug screening but also for disease modeling using patient-derived iPSC (19) (Figure 1).

The multicellular nature of the lung can also be recapitulated using IPSC-derived organoids (20) allowing generation of 3D structures containing lung progenitors, alveolar type 2 (AT2) cells as well as airway cells and alveolar macrophages (21). In this field, the organoid technology has been used to model hereditary lung diseases such as cystic fibrosis with demonstration of gene correction potential (22).

It therefore appears that the unique pluripotent nature of iPSC is a major asset for the generation of organoids-in-a dish toward any types of structures with advantages but also disadvantages as compared to adult-tissue-derived organoids, as summarized in Table 1.

**Table 1:** Comparative features of adult tissue vs. iPSC-derived organoids.

**Origin of organoid**	**Availability**	**Protocols**	**Differentiation potential**	**Potential** use as ATMP^*^	**Cancer** ** organoid** ** models**	**Utility in** ** cancer immunology research**	**Potential** ** for use in infectious diseases**	**Genome** ** editing**	**Organ-on** ** chip studies**	**Potential biobanks**	**Challenges** ** ahead in** ** 2021**
Adult-tissue-derived	Requires tissue and biopsy	Relatively easy	Depends on the type of organs and stem cells	Limited	High potential	Yes, TME^**^ with immune components are present	Limited (Requires tissue sampling)	Limited	Yes	Yes	Improved differentiation protocols
											Ability to expand
iPSC-derived	Readily available from iPSC	Complex and multistep procedures	Theoretically unlimited towards any tissue	High potential	High potential but TME absent.	TME components are not reproduced in a single step	High Potential	High potential	Yes	Yes	Simplified differentiation protocols
					Interest in hereditary cancer studies						Adequate vascularization and innervation

* *Advances therapeutic medicinal product*.

** *Tumor micro-environment*.

Finally, this highly sophisticated technology with several steps of culture may hold promise not only for the study of infectious diseases (such as SARS-Co-V2) but also for therapeutic purposes as an ATMP product (see below).

## Organoids as ATMPs: Hype Or Hope?

Given the differentiation potential of iPSC toward almost any organoids, the next question is their potential use as ATMPs. Large scale cGMP grade production of organoids could also lead to the possibility of manufacturing “transplantable” organoids and tissues in the future. From this regard, cardiac and liver tissues could be candidates be generated for transplantation purposes. However, in this field, many efforts are underway and many obstacles remain to be solved. If the use of IPSC-derived corneal cell transplantation has already began in a trial in Japan (23), iPSC-derived organ transplantation in humans is currently a long way from clinical applications but there are studies showing the functional cells can be manufactured. Indeed, in experimental conditions, it has been shown that iPSC-derived kidney nephron structures improve acute renal failure in mice (24). Similarly, erythropoietin-(EPO)-producing iPSC)-derived nephrons could improve anemia associated with terminal kidney failure (25).

In lung diseases such as idiopathic pulmonary fibrosis where the only cure is the lung transplantation, the possibility of generating and transplanting iPSC-derived autologous alveolar epithelial cells could have a significant impact on the prognosis (26).

In the field of diabetes, the transplantation of iPSC-derived islet organoids could be of therapeutic interest in the future as this has been validated in experimental setting (27).

What are the challenges lying ahead before the clinical applications? The vascular organization of future organoids is a major challenge but from patient-specific IPSC, it could be possible to generate after imprinting a brain or heart organoid containing microvasculature derived from HUVEC cells (28).

Similarly, it is necessary to provide in the organoids of the future an adequate innervation system. The possibility of generating iPSC-derived intestinal tissues with an enteric nervous system has been described, generated by combing human intestinal organoids and pluripotent-stem cell derived neural crest cells (29). A combined IPSC-derived hierarchized organoids called “assembloids” have also been described in the hepatobiliary system, generated by inducing the fusion of anterior and posterior gut spheroids, leading a hepato-biliary pancreatic organoids (30). Transplantation of these structures into immunodeficient mice failed however, to give rise to a multi-organ differentiation (30).

## iPSC-Derived Organoids in Cancer

In the field of cancer, the possibility reproducing cancer of a given organ could be of substantial interest, especially to develop drug screening and for precision medicine. From this regard, cancer organoids could allow to capture genetic heterogeneity as well as the progression-related modifications in a given cancer. Using adult-tissue derived organoids, several studies have shown that organoids generated from the initial tumor as well as from their metastatic counterparts match closely with the original tumor in breast cancer (31). Established from the initial tumor biopsies before any therapy, these organoids arising *in vitro* within 1–3 months could serve as an *in vitro* drug screening tools (32). One major drawback of this technology is the fact that it requires obviously the availability of a tissue sample which is not always possible. The growth of the structure is also limited as the tumor biopsy does not always recapitulate the hierarchical subtypes of a tumor. On the other hand, the major advantage of this approach as compared to IPSC-derived modeling is the possibility to capture the cellular components of the tumor microenvironment, including immune competent and immune-suppressive cells which could allow the potential responses to immune therapies such as check-point inhibitor therapies (see Table 1).

**Figure 2:**
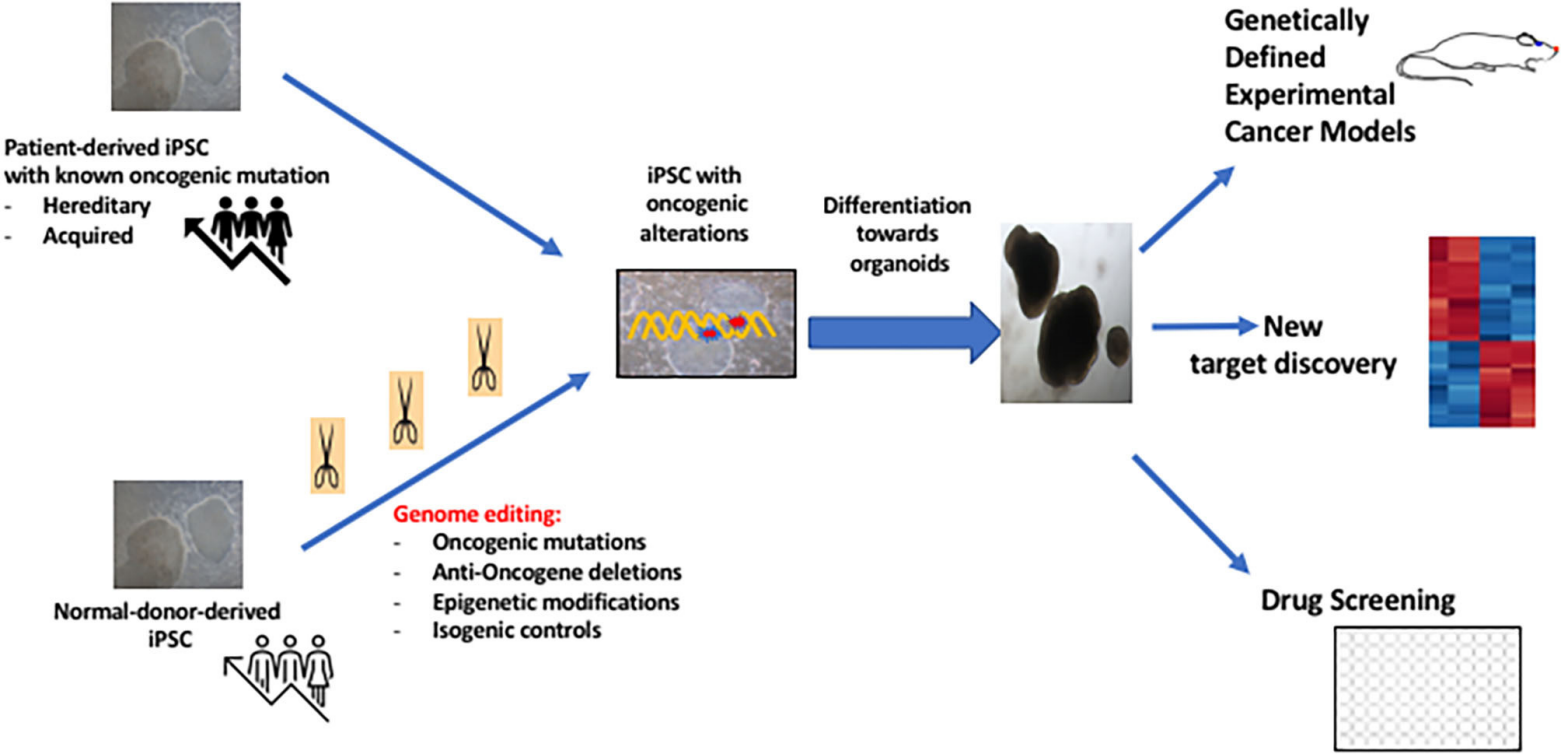
Potential interests of iPSC-derived cancer organoids in Cancer Medicine.

Although more challenging as compared to adult tissue-derived cancer derived organoids, iPSC-generated cancer organoids can be of interest in the study of patients with hereditary cancers. iPSC technology allows also the *de novo* generation of cancer organoids using genome editing (Figure 2). In the unique situation of the context of hereditary cancers, especially in the carriers of the oncogenic mutation with no established cancer, it may be possible to generate iPSC and to use this oncogene-bearing cell line as an organoid specific of the target tissue such as breast cancer or kidney cancer. This approach was first reported by the group of I. Lemischka using IPSC derived from patents with Li-Fraumeni disease (33).

In our group, we have generated c-MET-mutated iPSC from a patient with hereditary papillary renal cell carcinoma (34). We have shown that kidney organoids generated *in vitro* recapitulate the transcriptomic features of the primary PRCC of a large cohort of patients. The target genes that we have identified have been also confirmed in the kidney biopsies of patients with PRCC (34). The presence of a given oncogenic mutation in an iPSC line, allows the evaluation of the phenotype generated upon differentiation toward a given pathway and model therefore cancers occurring in several different tissue lineages. We have thus asked whether c-MET mutated iPSC could allow modeling glioblastoma, a tumor in which an overexpression of c-MET has been described in 10% of cases. We have showed that neural structures derived from these iPSC exhibit transcriptomic features close to that observed in human GBM (35).

The iPSC technology offers also the possibility of generating organoids after induction of specific genomic modifications using molecular manipulation of iPSC (Figure 2). An oncogenic mutation which can be induced in the pluripotent state, can then be propagated with generation of a “transformed organoid” as this has been shown for modeling glioblastoma (36). More recently, it has been shown that overexpression of KRAS G21D oncogene in pancreas acinar cells allow development of pancreas cancer *in vitro* (37) Similarly, recent work showed the possibility of generating in iPSC-derived RAS-mutated alveolar type 2 (AT2) cells, the induction of a genomic pathways similar to lung adenocarcinoma which is a driver gene in 30% cases (38). This study allowed to determine the early genomic changes occurring in the AT2 cells some of which were similar to transcriptomic features of in primary lung adenocarcinoma such as overexpression of Sox9 (38). Thus, iPSC-derived cancer organoid technology is expected to expand during the next decade, with several models developed for other cancers (39, 40) with increasing implication of microfluidic technology to study drug sensitivity (41). These technologies will also benefit from the molecular analyses and bioinformatics techniques with discovery of novel targets leading to therapeutic intervention (34, 42).

## Use OF iPSC-Derived Organoids in Infectious Diseases

Organoids appear today as major experimental tools for determining target cells and pathophysiology of viral infections. This concept has been successfully applied to Zika virus (43–45) and more recently to SARS-Cov-2 infections. In fact, in a very short period of time, the availability of these bioengineering tools led to their exponential use for several research teams worldwide to identify target cells for COVID-19 entry, and to evaluate the potential therapeutics, and vaccine approaches.

Using a large number of organoids derived from iPSC, it has been shown that the organoids such as pancreas are highly permissive to the virus (46).

Tiwari et al. have generated iPSC-derived brain and lung organoids to study the virus entry into different cell populations and their differential responses to COVID-19 infection (47) In particular they have shown that as compared to lung organoid derived cells, neural progenitors and astrocytes expressing low levels of ACE2 were not permissive to COVID-19, a finding that has been documented in pluripotent stem cells (47). The iPSC-organoid technology represents also a major tool to study drugs that best allow inhibition of virus entry into cells of different origin (48). Human IPSC-derived organoid technology allows also, by the combined use of CRUSPR CAS9 techniques, to determine the genetic susceptibility of human populations to Covid_19 (49). Indeed a unique sNP found in the 3′UTR of the Furin gene has been shown to influence the infection of lung and neuronal cells by Covid 19. These technologies could therefore help to define populations which could be an increased risk for Covid 19 infection (49). One other interesting potential use of organoid technology in the field of infectious diseases is the use of gastric organoids to study the infection by Helicobacter Pylori (50, 51).

## Perspectives and Challenges Ahead

Organoid technology, developed initially from the primary normal or diseased tissues/organs has achieved a novel major perspective by the use of iPSC technology, which offers the possibility of genomic editing and theoretically an unlimited proliferation and differentiation potential. As summarized, the technology holds tremendous potential but several hurdles remain, explaining its current limitations. For larger medical applications, better differentiation protocols are needed. The fact that cells organize themselves in 3D conditions suggest that some cell to cell interactions must occur to lead to pre-organoid structures called ‘aggregates” and to the phenomenon of symmetry breaking which occurs during normal embryonic development.

One other limitation of iPSC-derived organoids is the fact that they do not represent the typical environments which are usually found in tissues, especially in cancer in which a particular immune-suppressive microenvironment is present. Similarly, the application of this organoid technology in the future kidney transplants, will require the possibility of generating a functional vasculature but also a urine drainage system, which is not yet been achieved.

Thus, the generation a functional vasculature within the *in vitro* generated organoids is a significant challenge. Similary, organoids might miss some changes related to aging of the organ especially when generated from iPSC. A recently described human Organoid Atlas could be of major help to generate a “catalog” for human organoids including the standardization of their methodology. Single cell transcriptome analyses as well as spatial profiling will be of major areas of research during the next decade.

A H2020 project is currently in progress project within the human cell atlas project (https://hca-organoid.eu).

Finally some ethical issues might need to be discussed in the future with regard to the generation and the use of reprogrammed cells but also with regard to the creation of complex and increasingly sophisticated iPSC-derived human organoids (52). Indeed, such ethical issues will need to be discussed for example, with the possibility of generating complex brain organoids, complex integrated systems or early developmental structures such as amniotic sacs (53). These complex integrated systems have already been developed using microphysiological systems allowing organoids to be used for emulation of human biology in “human-organs-on-chips” systems and will pave the way for the drastic reduction of animal use for drug discovery experiments (54, 55).

Overall, both adult tissue and iPSC-derived organoids offer an unprecedented complementary information in almost all areas of medicine with accelerated discovery of novel targets and potentially as a therapeutic ATMP modality in the future.

## Author Contributions

All authors listed have made a substantial, direct and intellectual contribution to the work, and approved it for publication.

## Funding

This study was performed by the internal funds of INGESTEM.

## Conflict of Interest

The authors declare that the research was conducted in the absence of any commercial or financial relationships that could be construed as a potential conflict of interest.

## Publisher's Note

All claims expressed in this article are solely those of the authors and do not necessarily represent those of their affiliated organizations, or those of the publisher, the editors and the reviewers. Any product that may be evaluated in this article, or claim that may be made by its manufacturer, is not guaranteed or endorsed by the publisher.
